# Persistent Olfactory Dysfunction Three Years After COVID-19: A Multicenter Observational Study

**DOI:** 10.3390/pathogens15050541

**Published:** 2026-05-17

**Authors:** Xinyu Hu, Jingwen Li, Lin Chen, Hong Yu, Tao Zheng, Feng Dong, Xinyi Wang, Hanshu Liu, Qinwei Yu, Guiying Kuang, Tao Wang, Zhicheng Lin, Nian Xiong

**Affiliations:** 1 Department of Neurology, Union Hospital, Tongji Medical College, Huazhong University of Science and Technology, Wuhan 430022, China; 13508211092@163.com (X.H.); jingwenli1009@163.com (J.L.); wangxinyi_21@163.com (X.W.); liu1749676236@163.com (H.L.); wangtaowh@hust.edu.cn (T.W.); 2 Wuhan Red Cross Hospital, Wuhan 430015, Chinayuqinwei9@163.com (Q.Y.); 18071394079@163.com (G.K.); 3 Mclean Hospital, Harvard Medical School, Belmont, MA 02478, USA; zhicheng_lin@hms.harvard.edu

**Keywords:** COVID-19, olfactory dysfunction, anxiety, sleep disorder, observational cohort study

## Abstract

Olfactory dysfunction (OD) is a common sequela of SARS-CoV-2 infection, but its prevalence and associated factors beyond 2 years remain unclear. In this multicenter observational study, we assessed 155 recovered COVID-19 patients approximately three years after infection and included 170 age-matched healthy controls as a reference group. Demographic, clinical, psychological, and sleep-related data were collected through structured interviews. Olfactory function at the 3-year assessment was objectively evaluated using Toyota–Takagi (T&T) olfactometry. Paired baseline-to-follow-up T&T data were available only for a small exploratory subgroup of nine patients and were analyzed descriptively. At follow-up, 7 of 155 recovered patients (4.5%) met our T&T-based definition of persistent quantitative olfactory dysfunction, all of whom were older than 50 years. Emotional and sleep disturbances were also common, with descriptive trends toward higher frequencies among women and older individuals. Exploratory analyses suggested that insomnia (AIS > 6; OR 5.35, 95% CI 1.07–26.60; *p* = 0.033) and anxiety (HAMA ≥ 7; OR 10.54, 95% CI 1.21–91.82; *p* = 0.04) were associated with persistent T&T-defined quantitative OD, although the small number of outcome events limited statistical precision. These findings indicate that a small proportion of COVID-19 survivors have persistent objective OD 3 years after infection and that persistent OD is associated with anxiety and insomnia.

## 1. Introduction

Severe Acute Respiratory Syndrome Coronavirus 2 (SARS-CoV-2), the causative agent of Coronavirus Disease 2019 (COVID-19) [1], remains a major global health concern, with recent estimates suggesting approximately 2.3 billion COVID-19 cases worldwide annually [2]. While the public health emergency has ended, a significant proportion of survivors continue to experience long-term sequelae, collectively termed “long COVID” [3]. Among these, olfactory dysfunction (OD) is one of the most frequently reported and debilitating symptoms, profoundly affecting quality of life, nutritional intake, and emotional well-being [4,5,6].

The reported prevalence of OD during acute COVID-19 is unprecedented compared with previous viral pandemics for which systematic data are available [7], with the sudden onset of olfactory loss being a hallmark symptom [8]. The underlying mechanisms are thought to involve SARS-CoV-2’s tropism for the olfactory epithelium, mediated by the ACE2 receptor, leading to local inflammation and potential damage to olfactory sensory neurons [9,10]. More concerning, emerging evidence suggests that the virus may also affect central olfactory pathways, with neuroimaging studies revealing tissue damage in brain regions connected to the primary olfactory cortex [11]. This has raised concerns about the potential for long-term or even permanent olfactory impairment and its possible link to neurodegenerative processes, a phenomenon hypothesized to have occurred after the 1918 influenza pandemic [12].

While many patients recover olfactory function within weeks, a substantial subset reports persistent dysfunction. Previous studies have documented OD lasting up to two years post-infection [13,14,15]. However, the long-term objective olfactory outcomes beyond this timeframe remain insufficiently characterized. A recent large-scale study of US veterans identified loss of smell as a significant sequela up to three years after infection, but this and other studies have largely relied on subjective self-report or diagnostic codes rather than objective olfactory testing, and were limited to specific populations [3]. Furthermore, the factors influencing long-term recovery, such as demographic characteristics, psychological status (anxiety, depression), and sleep disturbances, remain incompletely characterized [16]. Recent reviews further emphasize that long COVID is a heterogeneous multisystem condition requiring individualized assessment and long-term management, but otorhinolaryngologic manifestations, including persistent OD, remain insufficiently characterized in long-term follow-up studies [17]. OD may not only contribute to psychological burden by impairing quality of life, dietary behavior, and safety perception but may also share overlapping neural circuits and inflammation-related mechanisms with anxiety, depression, and sleep disturbances [18,19]. Therefore, evaluating these factors may help clarify their potential association with long-term persistent OD. Understanding these factors is crucial for identifying at-risk individuals and developing targeted interventions for long-term olfactory rehabilitation.

Therefore, this multicenter observational study was designed to address these knowledge gaps. We assessed 155 recovered COVID-19 patients approximately three years after SARS-CoV-2 infection using objective Toyota–Takagi (T&T) olfactometry. In addition, paired baseline-to-follow-up T&T data were available for a small exploratory subgroup of nine patients; this subgroup was analyzed descriptively and was not considered representative of the full cohort. Our primary objectives were to determine the prevalence of persistent objective OD three years after SARS-CoV-2 infection and to identify demographic, clinical, psychological, and sleep-related factors associated with long-term olfactory impairment.

## 2. Materials and Methods

### 2.1. Study Design and Participants

This multicenter observational study included 155 patients who had recovered from COVID-19 before 1 April 2020 and 170 age-matched healthy controls. Patients were recruited from six hospitals in Wuhan, China, including Union Hospital, Jinyintan Hospital, Wuhan Red Cross Hospital, Central Hospital, Xinhua Hospital, and Fangcang Shelter Hospitals. All 155 recovered COVID-19 patients had been admitted to these designated hospitals or Fangcang shelter hospitals during the acute phase of infection. The inclusion criteria for recovery from COVID-19 in this study were as follows: (1) the patient must have maintained a normal temperature (≤37.3 °C) for more than three consecutive days; (2) respiratory symptoms such as cough, shortness of breath, and fatigue must have significantly resolved or subsided; (3) acute exudative lesions seen in chest computed tomography (CT) images must have shown substantial improvement, indicating recovery of lung function, and (4) the patient must have had two consecutive negative reverse transcription polymerase chain reaction (RT-PCR) test results, with each test separated by at least one day. The 170 healthy controls were age-matched individuals recruited during the same period from the same six hospitals and their surrounding communities, and were screened and assessed by the same research team. Eligible controls (1) had no history of COVID-19 or positive SARS-CoV-2 RT-PCR test results, and those without RT-PCR testing were required to have no COVID-19-related symptoms or known exposure history. (2) No previous olfactory dysfunction, and no history of chronic rhinosinusitis, nasal polyps, neurodegenerative diseases, or major psychiatric disorders that could affect olfactory function, psychological status, or sleep assessment. (3) Individuals with acute upper respiratory tract infection, acute nasal inflammation, or other conditions that could substantially affect olfactory function at the time of assessment were excluded. Healthy controls underwent all the same assessment protocol as recovered COVID-19 patients.

Before enrollment, verbal consent was obtained from each patient or from an accompanying relative when the patient was unable to provide consent. This study was approved by the Ethics Committee of Union Hospital, Tongji Medical College, Huazhong University of Science and Technology, Wuhan, China, on 20 February 2020, with written informed consent waived due to the rapid emergence of COVID-19, the urgent need for data collection, and the observational nature of the study (Approval No. [2020] Lun Shen Zi [0052]). All procedures were performed in accordance with the principles of the Declaration of Helsinki.

### 2.2. Data Collection and Clinical Assessments

Demographic characteristics, clinical symptoms, comorbidities, and treatment information were collected at baseline and at the 3-year follow-up through structured face-to-face interviews. When necessary, missing or unclear information was verified via telephone follow-up to ensure data accuracy.

### 2.3. Assessment of Psychological Status and Sleep Quality

Given recent evidence linking anxiety, depression, and sleep disturbances to post-COVID-19 olfactory dysfunction, we included these assessments to explore their potential associations with persistent OD. To evaluate psychological status and sleep quality, standardized and validated assessment scales were applied. Anxiety and depression were assessed using the Hamilton Anxiety Scale (HAMA) and Hamilton Depression Scale (HAMD), respectively. HAMA scores ≥ 7 and HAMD scores ≥ 7 were defined as indicating anxiety and depression, respectively [20]. As for the sleep condition, we used the Athens Insomnia Scale (AIS) and Pittsburgh sleep quality index (PSQI) to assess any sleep disturbances experienced by patients [21,22].

### 2.4. Olfactory Function Assessment

We used Toyota–Takagi (T&T) olfactometry scores system as an auxiliary diagnosis of dysosmia. This method consisted of five kinds of odors for olfactory sensitivity measurement. Odors included Garlic, Pineapple, Mint, Ginger and Rose. The concentration from low to high had eight stages: −2, −1, 0, 1, 2, 3, 4, and 5. An inspector held the standard gas test paper and gave it to the subject to smell in a non-smelly room, about 1 cm away from the nose. The concentration increased gradually from −2, and the recognition field, defined as the concentration when the patient identified the odor, was recorded on the measuring instrument. Each patient was tested twice, and the average of the recognition field was used to determine whether the olfactory function was normal or decreased. According to established T&T olfactometry criteria and previous validation studies in Chinese populations, a mean T&T recognition threshold > 1 was defined as quantitative olfactory dysfunction in this study [23,24]. All assessments were performed by trained examiners using the same standardized protocol. The main analysis of persistent OD was based on the T&T olfactory assessment conducted approximately three years after COVID-19 recovery. For a small exploratory subgroup, nine recovered COVID-19 patients had complete paired T&T olfactory data at both baseline and the 3-year follow-up, allowing a descriptive within-patient comparison. In these nine patients, baseline T&T testing had been performed during the acute phase of infection, within one week after hospital admission between January and March 2020, rather than before SARS-CoV-2 infection. Healthy controls underwent T&T olfactory testing during the same recruitment period and were assessed using the same testing protocol.

### 2.5. Statistical Analysis

All analyses were performed using IBM SPSS Statistics Version 24.0 (IBM Corp., Somers, NY, USA). The normality of continuous variables was assessed using the Shapiro–Wilk test. As continuous variables were not normally distributed, between-group comparisons were performed using the Mann–Whitney U test, while categorical variables were compared using the chi-square test or Fisher’s exact test, as appropriate. A value of *p* < 0.05 was considered statistically significant. Exploratory logistic regression was used to examine factors associated with persistent OD. Given the small number of persistent OD cases, no automated stepwise variable-selection procedure was used. Instead, the exploratory multivariable model was limited to variables that were significant in univariate analyses. The results were interpreted with caution because of the risk of model instability and overfitting.

## 3. Results

### 3.1. Basic Characteristics

In the recovered COVID-19 group, the median age of the recovered cases was 61 years (range, 27–79 years) and females accounted for the majority (65.2%). Among 155 recovered patients, 125 (80.6%) were over 50 years old. Ten (6.5%) patients had a smoking history, and all of them were over 50 years old. Median body mass index (BMI) of the 155 patients was 23.8 (within the normal range). The median duration of COVID-19 from symptom onset to hospital discharge was 30 days. A total of 87 (56.1%) patients had one or more symptoms at admission. The top three common symptoms at the onset of the illness were fever (78 [50.3%]), fatigue (72 [46.5%]) and cough (59 [38.1%]). In addition, 38 patients had shortness of breath after being infected, accounting for 24.5% (Table 1). Compared with healthy controls, recovered COVID-19 patients had a higher proportion of OD at the 3-year follow-up (4.5% vs. 0%, *p* = 0.005), whereas anxiety, depression, insomnia, and poor sleep quality did not differ significantly between the two groups (all *p* > 0.05; Table 1).

Comorbidities were observed in a subset of patients, with cardiovascular diseases being the most prevalent (25.8%), followed by endocrine system diseases (12.9%). The prevalence of respiratory, digestive, and nervous system diseases was each less than 10%. In addition, regarding treatments during the acute phase in 2020, nearly all patients received antiviral therapy. Furthermore, 97 patients (62.6%) were treated with Chinese herbal medicine (e.g., Lianhua Qingwen), 27 patients (17.4%) received systemic glucocorticoids, and 25 patients received both treatments (Table 2 and Table 3).

### 3.2. Prevalence and Characteristics of Persistent Olfactory Dysfunction

**Figure 1:**
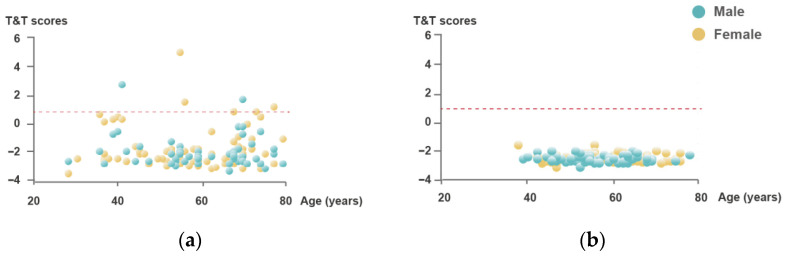
Age and gender distribution of olfactory function via T&T among the recovered COVID-19 patients (**a**) and controls (**b**). (**a**) The T&T scores for recovered COVID-19 patients are plotted against age, with gender indicated by color: females are shown in yellow and males in blue. A majority of the participants are concentrated in the age range of 50–70 years. Among recovered patients, seven participants (4.5%) still had impaired olfactory function. (**b**) The T&T scores for controls are shown in the same format, with a concentration of participants within the age range of 50–75 years. All participants had normal olfactory function.

All 155 patients who had recovered according to the criteria listed in Methods Section 2.1 underwent objective T&T olfactory assessment at the 3-year follow-up. At three years after SARS-CoV-2 infection, 7 of 155 patients (4.5%) met our T&T-based definition of persistent quantitative OD (Table 2). All seven patients with persistent OD were older than 50 years, suggesting an age-related vulnerability to long-term olfactory impairment. Among these patients, females accounted for the majority (71.4%), although no significant sex difference was observed (Figure 1). Additionally, none of the patients with persistent OD had a smoking history.

These findings indicate that although olfactory function recovered in most patients over time, a small subset of predominantly older individuals continued to exhibit persistent objective olfactory impairment up to 3 years after infection. The detailed odor-specific identification patterns of these seven patients are summarized in Table 4.

As an exploratory supplementary analysis, nine patients with complete paired T&T assessments at baseline and follow-up were described separately. Given the very small and selected nature of this subgroup, these findings should be interpreted only as descriptive and exploratory (Supplementary Table S1 and Figure S1). Their multisystem symptoms are summarized descriptively in Supplementary Table S2.

### 3.3. Emotional Disturbance and Sleep Disorder

**Figure 2:**
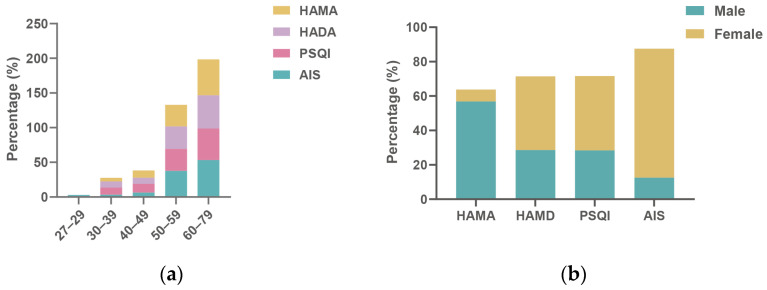
Age (**a**) and gender (**b**) distribution of psychologic and sleep disorders among the recovered COVID-19 patients. (**a**) The distribution of psychological and sleep disorders, including HAMA, HAMD, PSQI and AIS, is shown across different age groups. The percentage of patients reporting symptoms of anxiety, depression, and sleep disturbances increases with age, particularly in the older age groups (50–59 and 60–79 years), indicating a higher prevalence of emotional and sleep disorders in older individuals. (**b**) The gender distribution of psychological and sleep disorders among the recovered COVID-19 patients is shown. Female patients report higher percentages of anxiety, depression, poor sleep quality, and insomnia symptoms compared to their male counterparts, suggesting that females are more likely to experience emotional problems and sleep disorders post-recovery.

Given the potential relevance of psychological and sleep-related disturbances to persistent OD, we further assessed these features in the recovered COVID-19 cohort (Table 1 and Table 3). Overall, 58 patients (37.4%) exhibited anxiety symptoms and 77 (49.7%) showed depressive symptoms. Sleep disturbances were also common, with 32 patients (20.6%) meeting criteria for insomnia (AIS > 6) and 88 (56.8%) demonstrating poor sleep quality (PSQI > 5). Descriptively, emotional and sleep disturbances tended to be more frequent among older individuals and females (Figure 2), although not all comparisons reached statistical significance. These findings should therefore be interpreted as descriptive patterns rather than definitive subgroup differences. We further examined the associations of psychological and sleep-related features with persistent OD.

### 3.4. Factors Associated with Persistent Olfactory Dysfunction

To identify factors associated with persistent olfactory dysfunction, univariate analyses were performed across demographic, clinical, psychological, and sleep-related variables (Table 2 and Table 3). Among these, anxiety (HAMA ≥ 7, *p* = 0.011) and insomnia (AIS > 6, *p* = 0.034) were significantly more prevalent in patients with OD, whereas other variables showed no significant differences. Given the small number of outcome events, no automated stepwise variable-selection procedure was used. Anxiety and insomnia, which were significant in univariate analyses, were subsequently entered into an exploratory multivariable logistic regression model. In this model, anxiety (HAMA ≥ 7; OR = 10.54, 95% CI: 1.21–91.82, *p* = 0.041) and insomnia (AIS > 6; OR = 5.35, 95% CI: 1.07–26.60, *p* = 0.033) remained associated with persistent OD (Table 5). However, these estimates should be interpreted cautiously. These findings suggest that anxiety and insomnia may be associated with persistent olfactory impairment following COVID-19; however, the cross-sectional nature of the analysis precludes causal inference.

## 4. Discussion

In this multicenter observational cohort study with a 3-year follow-up of patients recovering from COVID-19, three principal findings were identified. First, 4.5% of patients met our T&T-based definition of persistent quantitative OD three years after infection, all of whom were aged over 50 years. Second, among the variables assessed in this study, anxiety and insomnia were associated with persistent OD, although this finding should be considered exploratory because of the small number of outcome events. Third, despite recovery from COVID-19 three years earlier, approximately one-third to one-half of patients continued to experience varying degrees of emotional and sleep disturbances at follow-up, with descriptive trends toward higher frequencies among women and older individuals. These findings suggest that although olfactory function recovers over time in most patients, a subset continues to experience persistent olfactory impairment accompanied by neuropsychological disturbances during long-term follow-up.

Notably, no cases of complete anosmia were observed in our cohort. This prevalence is markedly lower than the rates reported in previous shorter-term follow-up studies, including cohorts from Canada (32.2% at 11 months) [13], London (12.8% at 12 months) [14], and Italy (13.1% at 18 months) [15]. In addition, the longest follow-up study by Boscolo Rizzo et al. reported a prevalence of 11.8% at 2 years. However, these estimates should not be interpreted as directly comparable because the studies differed in design, participant characteristics, follow-up duration, olfactory assessment methods, and inclusion/exclusion criteria. Therefore, although the lower prevalence observed in our cohort may be consistent with the gradual recovery of olfactory function over time, it may also reflect methodological and population differences across studies. On the other hand, age is a well-established determinant of olfactory function. Previous studies have shown that the prevalence of OD increases with age, with approximately 20–30% of individuals over 60 years exhibiting olfactory impairment [25]. In our cohort, the median age was 61 years, and 80.7% of participants were older than 50 years. Although age did not reach conventional statistical significance in the univariate analysis (*p* = 0.052), all seven patients with persistent OD were older than 50 years, suggesting that age-related vulnerability may still be clinically relevant. Given the small number of outcome events, we could not reliably determine the independent contribution of age in the exploratory regression model. Interestingly, the observed prevalence of persistent OD in our cohort was lower than the estimated prevalence of age-related olfactory impairment in the general elderly population. This finding suggests that COVID-19-related OD may be largely reversible over time, while the small proportion of persistent cases may reflect a combination of age-related vulnerability and post-infectious olfactory injury rather than COVID-19 alone. Although persistent OD was uncommon in our cohort, recent reviews of long COVID emphasize that recovery trajectories may vary across organ systems and that symptom-oriented, individualized follow-up remains important [17].

We further found that anxiety and insomnia were significantly associated with persistent OD, suggesting that these conditions may be interlinked through shared pathophysiological processes rather than reflecting a direct causal pathway from psychological or sleep disturbances to OD. This finding is consistent with previous studies reporting a high prevalence of anxiety and sleep disturbances in post-COVID-19 populations [26,27], as well as associations between these conditions and impaired olfactory function. The underlying mechanisms are likely multifactorial. On the one hand, olfactory impairment itself may negatively affect quality of life and daily safety (e.g., food identification and environmental awareness), thereby contributing to or exacerbating anxiety and sleep disturbances [18]. On the other hand, SARS-CoV-2 infection may simultaneously affect central neural networks involved in olfaction, emotional regulation, and sleep–wake control, leading to concurrent dysfunction across these domains. For example, the virus may enter the nervous system via receptors such as neuropilin-1 (NRP-1) [19], which is not only expressed in the olfactory system but is also widely distributed in brain regions involved in emotional and sleep regulation. In addition, infection-induced inflammatory responses, such as interferon signaling, may disrupt both olfactory receptor expression and central nervous system function, thereby impairing emotional and sleep homeostasis [28]. Although derived from an animal model, that study suggests a potential biological link between neuroinflammation, olfactory dysfunction, and anxiety-like behaviors. Furthermore, individual susceptibility factors, including genetic predisposition or chronic stress exposure, may contribute to the co-occurrence of these dysfunctions. It should be noted that the risk factor analysis in this study was based on cross-sectional data, and causal relationships between anxiety, insomnia, and OD cannot be established.

Alterations in olfactory function have been proposed as potential early features of neurodegenerative diseases. Olfactory dysfunction is commonly reported in early-stage Parkinson’s disease and Alzheimer’s disease [29]. In our cohort, all seven patients with persistent OD were older than 50 years, suggesting that age-related vulnerability may be relevant. However, 95.5% of patients had normal olfactory function at the 3-year follow-up, indicating that COVID-19-related olfactory impairment was largely reversible in most cases. Thus, persistent OD in this study should not be interpreted as evidence of neurodegenerative disease; any such link remains hypothesis-generating and requires validation in future studies with neurological follow-up, biomarkers, and neuroimaging.

Several limitations of this study should be acknowledged. First, the number of patients with persistent OD was small (*n* = 7), limiting statistical power and generalizability; the wide confidence intervals also indicate substantial uncertainty. Second, although objective T&T olfactometry was used, it may not capture all olfactory symptoms, such as parosmia, phantosmia, or mild impairment below the predefined cutoff. The absence of OD in healthy controls may also reflect selection bias toward healthier volunteers or the relatively conservative T&T cutoff. Third, the associated-factor analysis was cross-sectional, precluding causal inference. Fourth, recovery was defined according to clinical discharge/recovery criteria used during the early pandemic in 2020, which may not fully exclude residual abnormalities or multisystem long-COVID manifestations. In-person follow-up may also have underrepresented patients with severe long-COVID symptoms who were unable to attend onsite assessments. In addition, several potential confounders, including educational level, socioeconomic status, pre-existing psychiatric history, medication use, and other long-COVID sequelae, were not systematically assessed. Dichotomizing psychological and sleep scale scores may also have reduced information. Finally, because all patients were infected during the early Wuhan outbreak and data on reinfection, vaccination, and later variant exposure were unavailable, the findings may not be directly generalizable to vaccinated populations or infections caused by later variants.

This study also has several notable strengths. First, the observational design and multicenter cohort enhance the reliability and representativeness of the data. Second, the 3-year follow-up duration represents one of the longest follow-up periods reported to date in studies of COVID-19-related olfactory dysfunction. Third, objective olfactory assessments were performed at two defined time points, namely during the acute phase of infection and at the 3-year follow-up, improving the accuracy of olfactory outcome assessment.

## 5. Conclusions

In this multicenter observational study, most recovered COVID-19 patients had normal objective olfactory function at the 3-year follow-up, whereas a small proportion continued to meet our T&T-based definition of persistent quantitative OD. Persistent OD was observed only among individuals older than 50 years. Anxiety and insomnia were associated with persistent OD in exploratory analyses, highlighting the importance of assessing psychological and sleep-related conditions during long-term follow-up. These findings support the continued monitoring of COVID-19 survivors, particularly older individuals, with attention to olfactory function and broader neuropsychological outcomes. Future studies with larger sample sizes, repeated longitudinal assessments, and broader evaluation of long-COVID sequelae are warranted.

## Figures and Tables

**Table 1:** Basic information of participants.

Characteristics	Patients	Control	*p* Value
	(*n* = 155)	(*n* = 170)	
Demographics			
Female, *n* (%)	101 (65.2)	99 (60.5)	0.358
Male, *n* (%)	54 (34.8)	71 (41.8)	0.358
Age, median (IQR), years	61.0 (16.0)	60.0 (11.0)	0.103
BMI, median (IQR), kg/m^2^	23.8 (3.0)	23.7 (2.8)	0.332
Smoking history, *n* (%)	10 (6.5)	7 (4.5)	0.482
Olfactory function			
T&T, median (IQR)	−2.4 (1.0)	−2.8 (0.4)	<0.001 *
T&T > 1	7.0 (4.5)	0	0.005 *
Psychologic and sleep condition			
HAMA, median (IQR)	6.0 (1.0)	5.0 (2.0)	0.867
HAMD, median (IQR)	7.0 (1.0)	6.0 (3.0)	0.852
PSQI, median (IQR)	6.0 (6.0)	5.0 (2.0)	0.581
AIS, median (IQR)	3.5 (5.0)	4.0 (3.0)	0.052
HAMA ≥ 7, *n* (%)	58 (37.4)	55 (32.4)	0.338
HAMD ≥ 7, *n* (%)	77 (49.7)	73 (42.9)	0.180
PSQI > 5, *n* (%)	88 (56.8)	83 (48.8)	0.152
AIS > 6, *n* (%)	32 (20.6)	33 (19.4)	0.781
COVID-19 related symptoms			
Duration, median (IQR), days	30 (22.3)	/	
Chest pain	11 (7.1)	/	
Fatigue	72 (46.5)	/	
Cough	59 (38.1)	/	
Rhinorrhea	8 (5.2)	/	
Fever	78 (50.3)	/	
Pharyngalgia	40 (25.8)	/	
Shortness of breath	38 (24.5)	/	
Headache	31 (20.0)	/	
Myalgia	43 (27.7)	/	
Dizziness	20 (12.9)	/	
Abdominal pain and diarrhea	30 (18.4)	/	
Nausea and vomiting	25 (16.1)	/	
More than two system symptoms	87 (56.1)	/	

* *p* < 0.05; COVID-19, coronavirus disease 2019; IQR, Inter-Quartile Range; T&T, Toyota–Takagi olfactometry scores; HAMA, Hamilton Anxiety Scale; HAMD, Hamilton Depression Scale; PSQI, Pittsburgh sleep quality index; AIS, Athens Insomnia Scale.

**Table 2:** Demographic, COVID-19-related symptoms, and treatment factors associated with persistent OD in recovered COVID-19 patients (*n* = 155).

Variants	T&T > 1	T&T ≤ 1	Fisher’s Exact Test	*p* Value
Demographics				
Gender, Female, *n* (%)	5 (71.4)	96 (64.9)	0.103	0.550
Age, median (IQR), years	65 (12.0)	61 (16.0)	N/A	0.052
BMI, median (IQR), kg/m^2^	23.9 (3.6)	23.8 (3.0)	N/A	0.911
Smoking history, *n* (%)	0	10 (6.8)	0.452	0.652
Duration, median (IQR), days	29 (23.0)	30 (22.3)	N/A	0.361
COVID-19 related symptoms				
Cardiovascular system	1 (14.3)	72 (48.6)	0.942	0.284
Chest pain	0	11 (49.3)	0.942	0.284
Fatigue	1 (14.3)	71 (46.6)	0.877	0.296
Respiratory system	5 (71.4)	95 (64.2)	0.093	0.525
Cough	2 (28.6)	57 (37.2)	0.093	0.525
Rhinorrhea	0	8 (5.4)	0.399	0.685
Fever	5 (71.4)	73 (48.6)	0.090	0.536
Pharyngalgia	1 (14.3)	39 (25.7)	0.460	0.437
Shortness of breath	1 (14.3)	37 (25.0)	2.299	0.142
Nervous system	1 (14.3)	51 (34.5)	0.038	0.569
Headache	1 (14.3)	30 (53.3)	0.297	0.435
Myalgia	1 (14.3)	42 (27.0)	0.008	0.611
Dizziness	2 (28.6)	18 (12.2)	0.028	0.607
Digestive system	0	31 (20.9)	0.991	0.641
Abdominal pain and diarrhea	0	30 (19.6)	0.121	0.685
Nausea and vomiting	2 (28.6)	23 (16.2)	0.018	0.315
More than two system symptoms	3 (42.9)	25 (16.9)	0.524	0.365
Treatment during acute infection				
Chinese herbal medicine	5 (71.4)	92 (62.2)	0.052	0.554
Glucocorticoid	1 (14.3)	26 (17.6)	0.050	0.648
Integrated both methods	1 (14.3)	24 (16.2)	0.018	0.685

Data are presented as *n* (%) or median (IQR). *p* values were calculated using the Mann–Whitney U test for continuous variables and Fisher’s exact test or chi-square test for categorical variables, as appropriate. These analyses were exploratory because of the small number of persistent OD cases. COVID-19, coronavirus disease 2019; OD, olfactory dysfunction; T&T, Toyota–Takagi olfactometry; BMI, body mass index; N/A, not applicable.

**Table 3:** Psychological/sleep-related variables and comorbidities associated with persistent OD in recovered COVID-19 patients (*n* = 155).

Variants	T&T > 1	T&T ≤ 1	Fisher’s Exact Test	*p* Value
Psychological and sleep symptoms				
HAMA ≥ 7	6 (85.7)	52 (35.1)	7.302	0.011 *
HAMD ≥ 7	4 (57.1)	73 (49.3)	0.163	0.493
PSQI > 5	6 (85.7)	82 (55.4)	2.502	0.115
AIS > 6	4 (57.1)	28 (18.9)	5.961	0.034 *
Comorbidities				
Cardiovascular system	3 (42.9)	37 (25.0)	0.065	0.547
Endocrine system	2 (28.6)	18 (12.2)	0.028	0.607
Respiratory system	1 (14.3)	7 (4.7)	0.002	0.722
Nervous system	0	2 (1.4)	0.295	0.754
Digestive system	1 (14.3)	9 (6.1)	0.452	0.652
More than two systems	3 (42.9)	26 (17.6)	0.547	0.370

Data are presented as *n* (%) or median (IQR). *p* values were calculated using the Mann–Whitney U test for continuous variables and Fisher’s exact test or chi-square test for categorical variables, as appropriate. These analyses were exploratory because of the small number of persistent OD cases. COVID-19, coronavirus disease 2019; OD, olfactory dysfunction; T&T, Toyota–Takagi olfactometry; HAMA, Hamilton Anxiety Scale; HAMD, Hamilton Depression Scale; PSQI, Pittsburgh Sleep Quality Index; AIS, Athens Insomnia Scale. * *p* < 0.05.

**Table 4:** Olfactory dysfunction of the recovered patients by T&T (*n* = 7).

Characteristics	No. (%)
T&T > 1	7 (4.5)
Gender	
Male	2 (28.6)
Female	5 (71.4)
Age	
50–59	2 (28.6)
60–69	1 (14.3)
70–79	4 (57.1)
Abnormal odors	
Garlic	5 (71.4)
Pineapple	7 (100.0)
Mint	5 (71.4)
Ginger	7 (100.0)
Rose	7 (100.0)

T&T, Toyota–Takagi olfactometry scores.

**Table 5:** Exploratory multivariable logistic regression analysis of factors associated with persistent olfactory dysfunction (*n* = 155).

Variants	Odds Ratio (95% CI) ^#^	*p* Value
HAMA ≥ 7	10.54 (1.21–91.82)	0.041 *
AIS > 6	5.35 (1.07–26.60)	0.033 *
Age		0.053
Gender		0.866
BMI		0.736
Smoking history		0.449
Respiratory system disease		0.492

**^#^** This exploratory multivariable logistic regression model included anxiety and insomnia, which were significant in univariate analyses. No automated stepwise variable-selection procedure was used. The estimates should be interpreted with caution because only seven patients had persistent OD. BMI, Body Mass Index; HAMA, Hamilton Anxiety Scale; AIS, Athens Insomnia Scale. * *p* < 0.05.

## Data Availability

All data supporting the reported results are available in the original studies included in the article and are shown in the figures and tables included in the present manuscript.

## Supplementary Materials

The following supporting information can be downloaded at: https://www.mdpi.com/article/10.3390/pathogens15050541/s1, Supplementary Table S1. Demographic and clinical characteristics of the exploratory subgroup (*n* = 9) with paired T&T olfactory assessments at baseline and 3-year follow-up. Supplementary Table S2. Descriptive summary of multiorgan sequelae in the exploratory subgroup (*n* = 9) with paired T&T olfactory data. Supplementary Figure S1. Longitudinal changes in T&T olfactory scores from baseline (acute infection) to 3-year follow-up in the exploratory subgroup (*n* = 9). At baseline (acute infection, 2020), 7 of the 9 patients had normal olfactory function, and 2 patients (Patients 3 and 6) had olfactory dysfunction (T&T > 1). At the 3-year follow-up, all nine patients showed normal olfactory function. This figure is descriptive and exploratory; no inferential statistics were applied.
