# Supplementary material for: Persistent Olfactory Dysfunction Three Years After COVID-19: A Multicenter Observational Study
Source: Pathogens. 2026 May 17;15(5):541. doi: 10.3390/pathogens15050541 (PMC13209864; doi:10.3390/pathogens15050541)
Supplement: Supplementary file 1 [file pathogens-15-00541-s001.zip › pathogens-4303244-supplementary.pdf]

Supplementary Table S1. Demographic and clinical characteristics of the exploratory subgroup (*n*=9) with paired T&T olfactory assessments at baseline and 3-year follow-up.

Patient	Age	Sex	Duration (Days)	BMI	Underlying diseases	Symptoms	Interventions
1	51	F	38	22.9	Rhinitis, diabetes，cardiovascular disease	Cough, fever, pharyngalgia, abdominal pain and diarrhea, dyspnea, fatigue, myalgia, stethalgia	LHQW
2	67	F	16	21.5	/	/	/
3	62	M	48	23.5	/	Abdominal pain and diarrhea, dyspnea	LHQW
4	59	F	29	24	Rhinitis	/	/
5	67	F	14	21.5	/	/	/
6	71	M	35	25	Rhinitis	/	/
7	35	F	11	23.9	/	Cough, rhinorrhea, fever, headache, pharyngalgia, abdominal pain and diarrhea, dyspnea, fatigue, myalgia	LHQW
8	52	F	24	22	/	Cough, headache, pharyngalgia, fatigue, myalgia	LHQW
9	51	F	20	25.3	/	Cough, fever, pharyngalgia, fatigue, myalgia	LHQW

This subgroup was exploratory and not representative of the full cohort. No inferential statistics were performed.

BMI, Body Mass Index; LHQW, Lianhuaqingwen medicine.

Supplementary Table S2. Descriptive summary of multiorgan sequelae in the exploratory subgroup (*n*=9) with paired T&T olfactory data.

Case	Gender	Pre-existing disease			Hospitalization (days)*	Organ of sequelae					Number of organs involved#
		Respiratory system	Cardiovascular system	Endocrine system		Cardiovascular system	Respiratory system	Nervous system	Digestive system	Psychologic and sleep system	
1	M				48		Yes		Yes	Yes	3
2	F				11	Yes	Yes	Yes		Yes	4
3	F	Rhinitis	Hypertension	Diabetes	38	Yes	Yes	Yes	Yes	Yes	5
4	F				20	Yes	Yes	Yes		Yes	4
5	F				24	Yes	Yes	Yes		Yes	4
6	F				49		Yes	Yes	Yes	Yes	4
7	F				46	Yes		Yes		Yes	3
8	F		Hypertension	Diabetes	46	Yes	Yes	Yes	Yes	Yes	5
9	F				53		Yes	Yes		Yes	3

The data were collected at the 3-year follow-up. All nine patients had recovered olfactory function at follow-up (Supplementary Figure S1). The table is descriptive only; no statistical comparisons were made.

* all had mild COVID-19

[#] anxiety: HAMA≥7;   depression: HAMD≥7; sleep disturance: PSQI>5; Insomnia: AIS

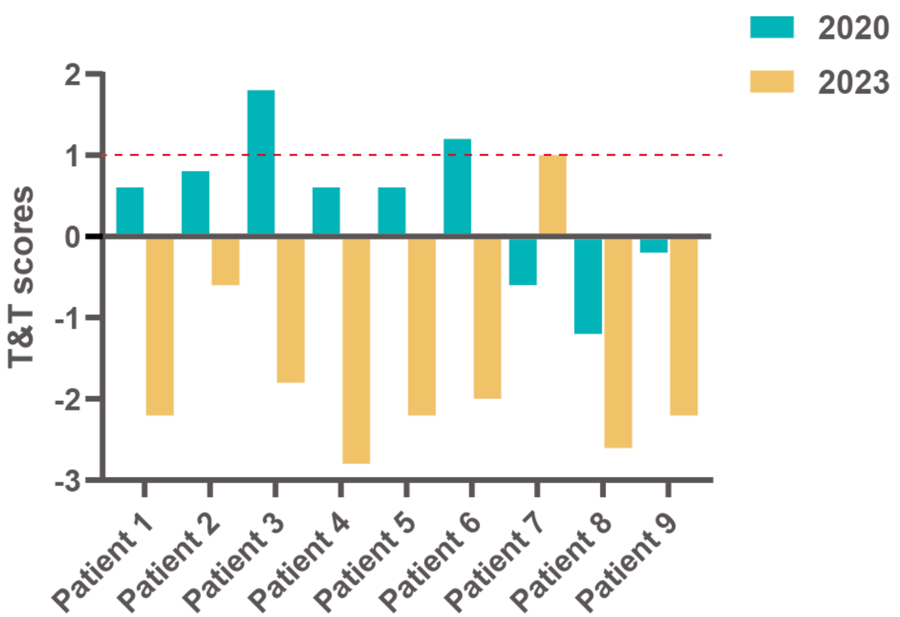

Supplementary Figure S1. Longitudinal changes in T&T olfactory scores from baseline (acute infection) to 3-year follow-up in the exploratory subgroup (*n*=9). At baseline (acute infection, 2020), 7 of the 9 patients had normal olfactory function, and 2 patients (Patients 3 and 6) had olfactory dysfunction (T&T >1). At the 3-year follow-up, all nine patients showed normal olfactory function. This figure is descriptive and exploratory; no inferential statistics were applied.
